# Clarifying Research Article Classifications: A Proposal for Defining Original Studies, Systematic, Scoping, Integrative, and Narrative Reviews, and Case Reports

**DOI:** 10.7759/cureus.94465

**Published:** 2025-10-13

**Authors:** Joe Iwanaga, Norio Kitagawa, R. Shane Tubbs

**Affiliations:** 1 Department of Neurosurgery, Tulane University School of Medicine, New Orleans, USA; 2 Department of Oral and Maxillofacial Anatomy, Institute of Science Tokyo, Tokyo, JPN

**Keywords:** category, journal, medicine, original article, review article, science, taxonomy

## Abstract

The academic publishing landscape increasingly demands precision in research reporting and article classification. However, confusion persists over the distinctions between original studies and systematic, scoping, integrative, and narrative reviews, particularly when studies use secondary or aggregated data. This paper critically examines the defining features of each article type, highlights frequent misconceptions in peer review (e.g., the expectation for systematic data extraction in narrative reviews), and proposes a clear taxonomy based on methodological rigor and knowledge generation. We argue that originality should be defined by creating new knowledge, not by the exclusive use of primary data. Through literature examples and classification criteria, we call for harmonization across journals and editorial policies to improve clarity, transparency, and the integrity of scientific reporting.

## Editorial

In scholarly publishing, the distinctions among the types of research articles are often blurred, leading to inconsistent expectations among authors, reviewers, editors, and readers. This is particularly evident in clinical medicine, dentistry, and the anatomical sciences, where literature reviews and data syntheses are common but poorly categorized [1]. While systematic reviews and original studies have well-recognized roles, the emergence of scoping reviews and confusion around the methodological expectations of narrative reviews warrant renewed attention.

Current definitions and limitations

We review conventional definitions. An original article reports new data derived from the authors' experiments or observations and provides novel contributions to science [2]. A systematic review synthesizes existing studies using a predefined search strategy and inclusion criteria [3]. A scoping review maps key concepts and gaps in a field using systematic methods but without quantitative synthesis [4,5]. An integrative review integrates findings from diverse study designs, including quantitative, qualitative, and theoretical works, to generate new conceptual or theoretical insights [6]. A narrative review provides expert opinion or a broad overview without a structured methodology [7]. A case report is a detailed description of a single patient's clinical presentation, diagnosis, treatment, and outcome. A case series describes a group of patients (usually ≥2) with a similar diagnosis or treatment, without a control group. Recent publications have attempted to clarify these categories, but inconsistencies persist, particularly regarding secondary data analyses that generate new statistical outputs [8,9].

What makes a study 'original'?

The definition of an original study is often tied to the notion of "new data creation." Vintzileos and Ananth (2010) described how to write and publish it [10]. Traditionally, this has been interpreted as data generated through primary methods such as experiments, clinical trials, cadaveric dissections, or field observations. However, this definition is increasingly outdated. We argue that the essence of an original study lies not in the source of the data, whether primary or secondary, but in creating new knowledge through novel interpretation, synthesis, or statistical modeling, which is reproducible by other researchers. For instance, studies using large-scale secondary datasets (e.g., health registries, bibliometric databases, or published literature) can yield original findings by applying new analytical frameworks, developing predictive models, or revealing patterns not previously explored. Such studies contribute to the literature in ways comparable to traditional experimental designs.

This perspective aligns with contemporary academic writing standards, such as those outlined in Scientific Writing (2025), which propose the following criteria for identifying original research articles as primary sources [11]: The authors of an original research article are those who directly conducted the study, rather than summarizing or reviewing the work of others. The article presents a novel research hypothesis and systematically investigates it using established scientific methodologies. Both the methods employed and the results obtained are clearly and transparently reported. In some instances, the article is accompanied by supplementary materials that include the raw data collected during the study. These articles typically adhere to the IMRAD format (Introduction, Methods, Results, and Discussion) and include a concise and informative abstract.

Therefore, originality should be understood more broadly to include novel statistical or computational analyses of existing (secondary) data, the development of new theoretical models or conceptual frameworks, and the identification of previously unreported trends or relationships derived from rigorous methodology.

This inclusive view aligns with the evolving landscape of research, where access to data and the capacity for advanced analytics have transformed how knowledge is generated.

The problem with inappropriate classification

Many journals reject studies using secondary extracted data (e.g., bibliometric, regression-based meta-research) as non-original, despite their novel contribution. A review article is an academic publication that summarizes, analyzes, and assesses the current literature on a particular subject [12]. Reviews do not present original experimental data; rather, they provide a critical and comprehensive synthesis of existing research, offering insights or interpretations based on previously published studies [13]. Conversely, some reviewers incorrectly request PRISMA-level rigor from narrative reviews, failing to understand their descriptive and interpretive intents [3,14]. When reviewers misunderstand or misalign with these distinctions, it can shift the article's intended style and lead to structural inconsistencies. While narrative reviews follow a traditional format, they remain valuable resources for helping readers grasp the broader context of a topic [6,7]. If the methodology restricts how to collect the data, this could result in insufficient data collection due to the exclusion of necessary data. Therefore, narrative review and systematic review should be regarded as fundamentally different types of scholarly work.

Proposal: a function-based taxonomy

We propose a new framework for classifying academic articles that moves beyond traditional labels (e.g., "original" vs. "review") and instead classifies them according to their function and methodological structure. This taxonomy is organized along two conceptual axes (Table 1).

**Table 1:** Proposed Classification of Scholarly Articles

Source of Data	Article Type	Systematic Method	New Knowledge Created	New Classification	Traditional Classification
		Systematicity	Originality		
Primary Data	Primary Data Study	Yes	Yes	Original	Original
	Case Report/Series	No	Yes	Case report/Case series	Case report/Case series
Secondary Data	Secondary Data Study	Yes	Yes	Original	Review
	Systematic Review	Yes	No (summarizes evidence)	Review or systematic review	Review or systematic review
	Scoping Review	Yes	No (maps field)	Review or scoping review	Review or scoping review
	Integrative Review	Partly (structured but flexible)	Yes (conceptual synthesis)	Review or integrative review	Review
	Narrative Review	No	No	Review	Review

Systematicity

This refers to the degree to which a study follows predefined, transparent, and reproducible methods. Highly systematic studies include randomized controlled trials, systematic reviews, and meta-analyses, where protocols are often registered in advance. In contrast, narrative reviews or expert opinion pieces may rely on flexible or interpretive methodologies.

Originality

This denotes whether the article contributes new knowledge, data, insights, or theoretical constructs. A study may be considered original if it introduces primary data, novel methodologies, reinterpretations of existing datasets (e.g., secondary analysis), or new conceptual frameworks. Non-original articles include most reviews, commentary, or educational summaries, which primarily synthesize existing knowledge.

By combining these two axes, we categorize publications more meaningfully based on their scholarly contribution and methodological rigor, rather than on superficial labels or discipline-specific conventions.

Misclassification of article types leads to confusion, rejections, and reduced transparency in the scientific process. By redefining original research based on function and output rather than data origin, we can better align research classification with the realities of contemporary scholarship.
